# Issues with data transformation in genome-wide association studies for phenotypic variability

**DOI:** 10.12688/f1000research.2-200.v1

**Published:** 2013-10-02

**Authors:** Xia Shen, Lars Rönnegård

**Affiliations:** 1 Division of Computational Genetics, Department of Clinical Sciences, Swedish University of Agricultural Sciences, Uppsala, SE-750 07, Sweden; 2 Statistics, School of Technology and Business Studies, Dalarna University, Falun, SE-791 88, Sweden; 3 Division of Quantitative Genetics, Department of Animal Breeding and Genetics, Swedish University of Agricultural Sciences, Uppsala, SE-750 07, Sweden

## Abstract

The purpose of this correspondence is to discuss and clarify a few points about data transformation used in genome-wide association studies, especially for phenotypic variability. By commenting on the recent publication by Sun *et al.* in the *American Journal of Human Genetics*, we emphasize the importance of statistical power in detecting functional loci and the real meaning of the scale of the phenotype in practice.

## Correspondence

Recently, Sun *et al.* ^1^ raised an interesting suggestion concerning the use of variance-stabilization transformations in genome-wide association studies (GWAS) for phenotypic variability. Specifically, Sun *et al.* revisited Yang *et al.*’s ^2^ results on the variability-controlling locus *FTO* for human body mass index (BMI) and claimed that the underlying variability across genotypes might not be as large as Yang *et al.* had seen. Although it was an important point that Sun *et al.* discussed, especially when quantitatively studying phenotypic variability has become such a hot topic, it is our opinion that there are some issues with the transformation approach that Sun *et al.* proposed.

**Figure 1.**
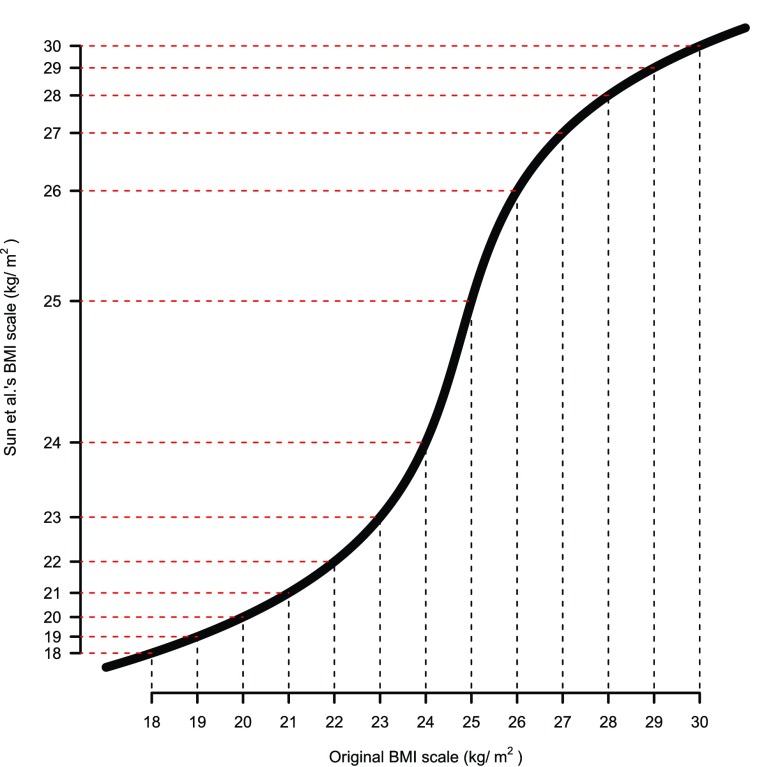
Comparison of the original scale of body mass index (BMI) and the transformed scale using Sun *et al.*’s ^1^ transformation. The transformation was determined by the phenotypic distribution across *FTO* genotypes reported by Yang *et al.* ^2^.

First of all, if we take Sun *et al.*’s transformation according to Yang *et al.*’s phenotypic mean and variance per *FTO* genotype class, *i.e.* a one-to-one map through an inverse hyperbolic sine function, the BMI scale will become rather different compared with the ordinary measurement that we normally use ( Figure 1). On the transformed scale of BMI, the difference between two persons who have a BMI of 24 and 25 kg/m ^2^ is much larger than that between two BMIs of 20 and 21 kg/m ^2^, which is strange in reality since the original BMI scale is what we commonly use and also what we care about. Sun *et al.*’s main argument here is that nearly all the measurement units are manmade. However, considering one of the traits of most interest, *e.g.* height, why should we regard the difference between 160cm and 170cm different from 170cm and 180cm? Although the definitions of most units can be arbitrary, some measurement scales do have meaning in real life.

Secondly, a key problem with Sun *et al.*’s transformation in practice is that such a transformation is marker-specific. Namely, when performing a GWAS, one needs to transform the phenotypic records differently for different markers, according to the phenotypic distribution across the genotypes per marker. This does not make much sense in practical analyses, because if there is a "best" scale of the phenotype, it should be used for all the markers across the genome, before testing the association between the phenotype and the markers. Using the tested marker to determine the transformation of the phenotype is strange. If a marker-specific transformation can be estimated, one should estimate a genome-specific transformation for GWAS, instead of doing different transformations marker-by-marker.

Thirdly, if the transformation of the phenotype is determined by one marker showing a significant effect on the phenotypic variability before testing the other markers, another significant effect on the phenotypic variability might be created due to such a transformation. In such a situation, it is problematic to decide which phenotypic scale we should choose.

Fourthly, several recent studies discussed that gene-gene or gene-environment interactions could cause significant variance heterogeneity across genotypes ^3– 6^, which makes testing variance-controlling loci a powerful tool to reveal potential interaction effects. Reducing the difference in variance across genotypes using a marker-specific variance-stabilization transformation would dramatically reduce such power. Regarding the biological sense of genetically regulated variance heterogeneity, empirical evidence has shown that a single causal locus could show a much higher significant effect on variance compared with the mean ^6^. In a particular population, such a locus may only be mappable through testing the variability rather than the magnitude of the phenotype.

The above issues cause us to question Sun *et al.*’s transformation in practice. The scale of the phenotype is certainly an important concern when interpreting an effect on phenotypic variability ^7^. However, one needs to be careful for the points above before applying any transformation on the data. In particular, the statistical power in detecting functional loci and the real meaning of the scale used should be emphasized.
