# Supplementary material for: Issues with data transformation in genome-wide association studies for phenotypic variability
Source: F1000Res. 2013 Oct 2;2:200. [Version 1] doi: 10.12688/f1000research.2-200.v1 (PMC3869493; doi:10.12688/f1000research.2-200.v1)
Supplement: Peer review history

# Peer review: Referee response for version 1

**Published:** 2013-11-01

**Authors:** Yurii Aulchenko, Yakov Tsepilov, Sodbo Sharapov

**Affiliations:** 1 Institute of Cytology and Genetics, Siberian Division of the Russian Academy of Sciences, Novosibirsk, Russian Federation; 2 Novosibirsk State University, Novosibirsk, Russian Federation

We agree with criticism raised by Shen and Ronnegard in their points 2 and 3 concerning the application of the transformation of Sun *et al*. in the context of whole-genome scans. Indeed, applying this transformation in SNP-specific manner is difficult to adopt conceptually. Sun *et al.* rightly suggest that “the scales on which we measure interval-scale quantitative traits are man-made and have little intrinsic biological relevance”, but the underlying intrinsic scale, and the function reflecting this scale into the observed, is likely to be unique and does not change with SNP. In that, the transformation applied to a trait should not change through the markers studied. Practically, this is not very difficult to implement, and as a simplest option one could think of the estimation of Sun’s transformation parameters from upper, middle and lower tertiles of the total phenotypic distribution. A more general approach (without restricting the data into three groups, but modelling the variance as a function of the mean) should be straightforward to implement.

We also understand the reasoning behind the Shen and Ronnegard’s points 1 and 4, but here we are less certain that the problem raised could be easily addressed. Specifically, one could argue with point 1 (“why should we regard the difference between 160cm and 170cm different from 170cm and 180cm?”): it is not that hard to imagine a biologically relevant model in which same changes of an “intrinsic scale” lead to different changes on the observed scale as the mean advances (an example would be Michaelis–Menten kinetics). Also both points 1 and 4 (losing power after transformation) relate not only to Sun *et al*.’s transformation, but to almost any transformation in wide use (e.g. Log, Box-Cox, Gaussenization/inverse-normal). While it is true that analysis of transformed trait may lead to reduced power (and specifically in case of Sun’s transformation applied in marker-specific manner to the analysis of variance heterogeneity it *should*), we have a feeling that one still would like to check whether the variance heterogeneity found can be modeled as a function of the mean (in which case any SNP affecting the mean is likely to show “control” of the variance as well).

Finally, we fully agree with comment of William Hill and Ian White who criticize Sun *et al*.'s statement that “‘In the absence of genotypic mean differences, we can hardly infer that differences in variances are *per se* of biological interest”. We think that the differences in variance *per se* are biologically and genetically plausible and interesting.

We have read this submission. We believe that we have an appropriate level of expertise to confirm that it is of an acceptable scientific standard.

# Peer review: Referee response for version 1

**Published:** 2013-10-07

**Authors:** William Hill, Ian White

**Affiliations:** 1 Institute of Evolutionary Biology, University of Edinburgh, Edinburgh, UK; 2 University of Edinburgh

Shen and Rönnegård (SR) comment critically and succinctly on the paper by Sun *et al*. published in AJHG which advocates that, before any claim of differences in variance among genotypes in a GWAS or similar study, a check should first be made whether these can be removed by a monotonic transformation. Each of SR’s four criticisms seems well justified. 

As 105 or more SNPs may be fitted in a GWAS study, what biological interpretation could be given to that number of different transformations or even on a limited subset of loci showing possible variance differences? If some loci give signals of mean but not variance difference, should these then be transformed to eliminate the scale effect on mean and perhaps reveal variance differences? Any concept of an original scale of measurement is lost, as SR point out. It is not obvious why the mere existence of a transformation designed to minimise differences in variance should prevent discussion of variance heterogeneity on the chosen scale. Equivalently, if we considered means of the three genotypes at the locus rather than just average effects, would our ability to transform the data at each locus such that heterozygotes were intermediate imply there was no dominance, or only that it was on a particular scale?

On a further point. Sun *et al*. (p395) comment: ‘In the absence of genotypic mean differences, we can hardly infer that differences in variances are *per se* of biological interest.’ That is to take too narrow a view: the mean and phenotypic variance (or CV) of a quantitative trait in any species take typical values, e.g. the CV for adult human height is ca. 4% and for BMI ca. 16% . There is direct evidence of genetic differences within species in environmental variance, with GWAS and other single gene studies, that cannot be removed by scale, so the level of the environmental variance is subject to evolutionary forces (e.g. Hill & Mulder 2010 Genet. Res. 92:381). To view variance as a biological phenomenon which is just some adjunct to the mean seems simplistic, as SR argue. Indeed one has to ask whether scale transformations have value unless there is a biological basis, such as a log transformation to account for multiplicative genetic effects; but that must then apply across all loci.

We have read this submission. We believe that we have an appropriate level of expertise to confirm that it is of an acceptable scientific standard.
